# Are Old Men Impotent? On a Sparse Discourse of Early Modern Medicine and Its Forensic Implications in Paolo Zacchia’s *Quaestiones medico-legales*

**DOI:** 10.3390/ijerph192416513

**Published:** 2022-12-08

**Authors:** Daniel Schäfer

**Affiliations:** Institute for the History of Medicine and Medical Ethics, Faculty of Medicine and University Hospital, University of Cologne, Joseph-Stelzmann-Str. 20, Geb. 42, D-50931 Cologne, Germany; ajg01@uni-koeln.de

**Keywords:** history, 17th century, infertility, male, erectile dysfunction, age groups, forensic medicine

## Abstract

In early modern medical literature, there are increasing references to sterility and impotence in older men. This is especially true of the *Quaestiones medico-legales* by the Roman physician Paolo Zacchia (1584–1659). In several books of this systematically structured manual, its author discusses medical and legal arguments on the one hand. On the other hand, in the 10th, only posthumously published volume, a total of five cases of impotence in old men are described on the basis of court decisions of the Rota Romana and expert opinions of the author. The paper examines these cases with regard to central statements on male impotence in old age, which are placed in the medical as well as the social and legal-historical context of the time. It becomes clear that old-age impotence and sterility were less a medical than a legal problem in the 17th century. However, the physician Zacchia emphasises the concept of biological age instead of historically transmitted numerical age limits. In this respect, his expert opinions already show the first signs of medical empiricism.

## 1. Introduction

Impotence and sterility in older age is a topic that occupies today’s medical community in various subdisciplines (urology, gynaecology, andrology, endocrinology, and fertility medicine). In older women, menopause and sterility as well as (in the case of the desire for children) overcoming them have been in the foreground up to now. In contrast, problems with sexual performance in particular dominate among older men, at least in anti-ageing medicine, and are often associated with so-called age hypogonadism and partial androgen deficit (PADAM syndrome) [1]. In principle, this sex constellation can be traced back to Greek and Roman antiquity: for example, the Corpus Hippocraticum addresses female infertility in a special writing *De sterilibus* [2,3]. References to male sterility, on the other hand, are rare in Greco-Roman medicine; instead, there are many indirect references to male impotence [4]. Information on age limits for fertility is found mainly in Aristotle. He set them at 45 to 50 years for women, and at 65 to a maximum of 70 years for men [5], (p. 140 (545 b27–30)). These age limits for the procreation of offspring are relatively frequently received, modified and sometimes also questioned in the late medieval and early modern medical literature. From the late 11th century onwards (Constantinus Africanus, *Liber de Coitu*), the transmission of medical statements on male impotence also begins (mainly from the Islamic cultural sphere). However, specific references to potency problems of older men are almost completely missing.

This gradually changed in the early modern literature. In addition to the Aristotelian age limits mentioned above, humanistically educated scholars also find references to the relevant age-critical statements of classical antiquity (Plautus, Horace, Ovid, Juvenal, Maximianus) [6]. However, counter-examples for procreative capacity at a high age are also given. Overall, however, this was a rather sparse discourse (in contrast to the discussion on female sterility), in keeping with the general marginalisation of old age in the specialist literature of the time [7], (pp. 173–183). One medical author, however, clearly deviates from this tendency: the Roman physician Paolo Zacchia (1584–1659), known in older medical history as the “founder of medical forensics” [8] and eponym of one of the oldest forensic journals still published today. Under Pope Innocent X, Zacchia acted as protomedicus for the entire Papal States as well as advisor and expert to the Rota Romana; this was the court for ecclesiastical legal decisions with regard to the entire Catholic Church, but also for civil legal issues of the then Papal States. Zacchia’s activity was also reflected in his main work, the nine volumes of *Quaestiones medico-legales* (published individually from 1621 to 1650). In these, numerous topics relevant to the ecclesiastical and secular jurisdiction of the time are discussed primarily from a medical perspective. However, Zacchia had also studied jurisprudence, and he systematically evaluated legal and medical literature for this purpose. These topics included male impotence in general and specifically in old age. The latter in particular is presented in the third and the ninth book of the *Quaestiones* [9,10]. As a physician influenced by Galenism, Zacchia elaborates different forms of impotence depending on the cause (organs involved and their humoral dyscrasias) and on the extent (reduced libido, erectile dysfunction, ejaculatory disorders, sterility). It should be borne in mind that at his time there was no knowledge of the composition of semen from prostatic secretions and motile spermatozoa of the testes.

It is now of particular interest that Paolo Zacchia’s nephew, Lanfranco Zacchia, published a tenth volume of the *Quaestiones* from his estate after his uncle’s death as part of a new complete edition [11]. This volume contains 85 of his uncle’s consultations (*consilia*; for a survey see [12]), one third of which, according to Silvia De Renzi, “are broadly related to sexuality and generation, including impotence, the right to inherit of a miscarried fetus, and the definition of conjugal rights” [13], p. 550. In addition, the tenth volume contains 100 legal decisions of the Rota Romana (*decisiones*), which Paolo Zacchia apparently collected and perhaps summarised for printing. Among them are five cases in which male age impotence plays a significant role.

In recent decades, a great deal of research has been presented on the history of old age and geriatrics in the early modern period [7,14,15,16,17,18]. Zacchia’s *consilia* have been investigated in various studies under different aspects [12,13,19]. More than a third of the *consilia* are now also available in English translations, among them Nos. 68 and 75 [20].

## 2. Materials and Methods

This article first examines (Section 3.1) five casuistics from the years 1603 to 1657, which were presented in the tenth volume of the Latin *Quaestiones* in a total of three *consilia* and five *decisiones*. With the help of the methods of qualitative literature analysis, central statements on male old-age impotence are elaborated and placed in the medical as well as the social and legal-historical context of the time. In particular, the following questions will be examined: In which social contexts did old-age impotence play an argumentative role in court? How was it determined and assessed by experts? What role did the reception of medical and legal literature play? In order to understand the legal-historical framework of the case histories, it is also necessary (Section 3.2) to briefly describe important canon law sources for the contemporary discussion on marriage annulment due to impotence.

## 3. Results

### 3.1. Casuistry Heard at the Rota Romana in the 17th Century

A critical reading of the *Quaestiones medico-legales* reveals five cases in which impotence due to old age played a significant role:

#### 3.1.1. Consilium 23

Paolo Zacchia examines the case of Aurelio Lingio, who died at the age of sixty and whose relatives brought an action against the right of inheritance of a son born six months after his death: the deceased had been impotent and incapable of procreation for a long time before his last illness due to joint complaints with fever and had revealed to his doctors at the time that he no longer had erections. The widow Artemisia, consequently accused of adultery, does not deny the ill condition of her former husband, but states that he had become capable of love again a few months before his death and had in turn “carnally recognised” her.

Zacchia, as a physician, judges that according to Aristotle, a sixty-year-old man is still capable of procreation without further ado and that Lingio had already proven himself to be very potent in earlier years by fathering various children by two wives and other concubines. The man’s arthritic condition did not necessarily permanently limit his procreative capacity; rather, the heated condition of the illness gave an indication of his abundant innate warmth: arthritics were generally particularly suitable for sexual intercourse and also willing to do so. Moreover, Artemisia was beautiful, experienced in the arts of love and had always slept in the same bed with her husband. Therefore, it was hardly conceivable that he would not have been sexually aroused despite the suffering. And finally, according to Hippocrates, a single coitus was enough to impregnate even twins and triplets [11], (pp. 175–177); [12], p. 162; [21], (pp. 52–53).

#### 3.1.2. Consilium 68

Whereas in the case of Lingio, the age and previous illness of the husband did not provide Zacchia with grounds for a finding of impotence, in another case he judges the other way around:

An unnamed sixty-year-old groom applies to the court for a vaginal examination of his bride to prove a constriction (*arctatio*) preventing coitus, as well as the prescription of a three-year probationary period for the marriage instead of an immediate annulment of the marriage. The latter had previously been requested by the virgin’s father because the man had “assaulted” her in vain for a month after the wedding and still had not been able to make her a wife. Three possible reasons for the groom’s impotence were given in the original complaint: his older age, a physical weakness, and a fistula in the perineal area (i.e., between the anus and the scrotum). Zacchia first presents arguments to refute these reasons (which were presumably in the husband’s counterclaim), and then refutes all of these refutals:

(1) The age of sixty or even seventy was not in itself a reason for assuming impotence, but this only applied to robust old men who were not weakened under the amount of their years and should not really be called old men at all. Conversely, fifty-year-olds who are already weak by nature could be old men, for, according to Galen [22], p. 387, ages are not determined by the number of years but by the existing powers; in this respect the husband is so weak that he is older than sixty. Therefore, he must not only be called an old man (*senex*), but even decrepit, and impotence must be assumed in such a one.
(2) The naturally existing physical weakness (*debilis*/*imbecillis*) was not pronounced as if the man was diseased (*valetudinarius*) in the sense of suffering against nature, but together with the age-related decrepitude it was an excellent and evident reason for impotence, especially in sexual intercourse with a virgin. Moreover, he had been plagued by long illnesses throughout his life, especially a weak stomach with poor digestion, so that he was diseased in this respect after all. A weak, cold stomach, moreover, is reckoned by all practitioners among the most prominent causes of impotence. This affliction, Zacchia said, would be aggravated in old age on account of the increasing coldness, for which reason a three years’ probationary period of marriage would be useless.
(3) Concerning the allegedly immaculate condition of the genitals, Zacchia refers to the surgeon’s report. The surgeon had found (apparently on the basis of an inspection) a penis of flaccid substance and considerable length; it was possibly hardly suitable for erection and hardening (necessary for defloration). It was thus clear that the obstacle to the consummation of the marriage lay entirely on the part of the man and was unchangeable; for there was a lack of inner warmth to remove this obstacle.
(4) The allegedly insignificant fistula was definitely above average; it was a remnant of repeatedly opened boils and had already tormented the man for many years. It constantly secreted foul-smelling pus in a remarkable amount, as stated in the surgeon’s report, and led to a cooling of the genitals. In view of these findings, it was superfluous to look for obstacles on the part of the bride. She had every right to reject her husband since he had tried to deflower her maliciously with his fingers [11], (pp. 295–297); [12], p. 158; [20].

#### 3.1.3. Decisiones 22, 26 and 27

Another but quite similar case is addressed in several older judgments of the Rota Romana (1603–1606). First, a couple was sentenced to the three-year probationary period after an inconclusive examination of their genitals, which, however, remained without “success”. Only afterwards (in *decisio* 27 of 28 April 1606) were the husband’s age (now approaching sixty) and his weak physical condition also taken as indications of possible impotence. The court therefore ordered a second examination of the wife by midwives regarding her virginity [11], p. 399.

#### 3.1.4. Decisio 59

This decision deals with the request of a certain Cosmo to reverse a gift made to his biological brother Marco Antonio during his lifetime. The condition of the donation in 1582 was that the brother marry and beget children “for the preservation of the house and the family”, which he had not done until now; and now he was sixty years old. The court decides that this request is rejected, among other things because according to Aristotle, Tiraqueau and Sánchez, procreative capacity can still be assumed at the age of sixty. In addition, the marriage prohibition of the ancient Lex Papia no longer applied, and the brother Marco Antonio still enjoyed lively health and constant strength [11], (pp. 458–460).

#### 3.1.5. Decisio 100 and Consilium 75

The last court decision recorded by Zacchia deals with a relatively recent case from 22 June 1657, and thus corrects two previous court decisions that are unfortunately not available. Two children of the prostitute Angela sue the Marchesa Maria, legitimate heiress of the deceased Giovanni Battista Veralli (Marchese Viscardi) for payment of alimony because the Marchese was their natural father. Witnesses appear on behalf of the Marchesa who claim that Angela revealed to Giovanni Jacopo Ferrari, a man of young, blooming age, that he was the natural father of the children. This was undoubtedly the case because Ferrari had also had “carnal” relations with Angela, loved her and, as it were, lived with her. On the other hand, the Marchese had already been seventy years old, and the law already excluded the assumption of procreation in the case of a sixty-year-old. The fact that the old man himself had mentioned paternity in many letters and had determined alimony was not decisive: he had been deceived by the prostitute with flattery; presumably, due to his poor state of health, he had not been in a position (legally?) to recognise an heir. For the same reason, the entry of the children in the baptismal register under the surname Verelli did not exclude the opposite, namely the assumption of a different paternity. The court agrees with this view of the defence and denies the children descent from the Marchese and the receipt of alimony. Decisive for this judgement is, among other things, the improbability of a procreation by the aged Marchese according to both “natural insight” and legal assumption, even if it could have occurred over a period of three years at Viscardi Castle—because other men and Ferrari in particular also had access to the children’s mother [11], (pp. 550–552). 

Interestingly, Zacchia’s *consilium* 75 takes this case even further: this expert opinion was obviously written for another, fourth trial in this matter, as it mentions the *decisio* of 22 June 1657 several times. Firstly we learn some additional details: that there were in fact two pregnancies (i.e., there was no twin birth), that the children have reddish hair like the 24-year-old Ferrari and that the boy resembles him, that the mother, the Venetian Angela Beverentia, only came to Rome for two years, that the Marchese began an affair with her at the age of 72 and died at 74, and that the woman, whom Zacchia also considers to be a prostitute, does not file suit for the children in Rome until 15 years after their birth. As in *consilium* 68, Zacchia first lists four main arguments of the plaintiff’s side and then refutes them. In *consilium* 75, too, he agrees that in principle 70-, 80- or even 100-year-olds could beget children; however, he emphasises that these could not be old or jaded men (*senes*), since the physical condition and not the years lived were decisive for this attribution. The Marchese, however, was slight, had a high, almost feminine voice and a bilious temperament, and was tormented all year round by catarrhs and many other symptoms. In particular, four cauterisations performed on the four extremities indicated a serious illness and reduced physical strength. Therefore, he must already have been an old man. In addition to biological age and disease, the therapeutic cauterisations themselves are also often the cause of impotence. Zacchia therefore recommends the rejection of the renewed complaint [11], (pp. 324–327); [20].

### 3.2. Framework of Canon Law

Some of the events, practices and names mentioned in the casuistics can only be understood against the background of legal framework conditions. In the course of the Middle Ages, a canon law discussion on impotence and infertility initially developed apart from medicine. Unlike the medical texts, it had its origins in concrete enquiries to church authorities about the validity or dissolution of a marriage. Furthermore, it placed a clear focus on male restrictions. Finally, as early as the 9th century (Hinkmar of Rheims), it introduced the distinction between natural (e.g., physical *frigiditas*, *infirmitas*) and supernatural causes (sorcery, *maleficium*) for impotence [23], p. 9; [24], (pp. 396–400). Permanent “coldness” (*frigiditas*) with consequent lack of copulation between the spouses was predominantly regarded as an impediment to marriage or as a possibility to have a marriage subsequently declared invalid; however, there were differing views on this among canonists [23], (pp. 62–73). There were essentially three obligatory conditions for annulment: physical completeness, temporal permanence and personal (i.e., existing towards each partner) absoluteness of impotence. If there was ambiguity regarding one of these conditions, an ecclesiastical court could order a probationary period of three years (*experimentum trienale*), following Roman law [23], p. 60. If the marriage was not consummated during this time, it could be annulled. Especially with regard to the determination of completeness, the practice of physical examinations of both spouses by sworn experts (surgeons, midwives, etc.) became established in the 13th century at the latest [25,26], (pp. 183–204).

Regarding age-related impotence and sterility, the *Glossa ordinaria* to the *Decretum Gratiani* (written by Accursius around 1230) was surprisingly unambiguous: “No one is so much an old man that he cannot sometimes become warm by nature or by means of aids. This is not the case with the impotent (*frigido*) or the child or the eunuch. And therefore, an old man is suitable for marriage. It is enough that he does not preclude [the procreation of] an heir” [27]. Around 1250, the theologians Albertus Magnus [28] and Thomas Aquinas indirectly corroborated this statement by also emphasising the differences between an old man and a *frigidus*; in doing so, they already made a clear distinction (apparently long before medicine) between *impotentia coeundi* and *impotentia generandi*: “As to the third objection [that old men are permitted to marry even though they are *frigidi*], it should be noted that old men, even if at some time they no longer have sufficient heat for procreation, nevertheless possess sufficient heat for carnal union. And it is permitted them to marry because it is a remedy [to them], though it doesn’t apply to them that it is a work of nature” [29]. From a theological as well as canonical point of view, therefore, the capacity for coitus was sufficient in old people; accordingly, even after the Fourth Lateran Council (1213), the consensus of the spouses and the initial consummation of the cohabitation were decisive for the validity of a marriage [30]. In many medieval cultures, the latter was also considered an essential condition for the beginning of a marriage, independent of canon law. If an age-related impotence occurred only afterwards, this was not considered by the canonists to be a legitimate reason for dissolving the marriage or having it declared invalid. For old people, marriage did not have to lead to the procreation of an heir, because its justification lay above all in the salutary, comforting support (*remedium infirmitatis*, *solatium humanitatis* [27]). For the rest, it was left open—in accordance with the state of medical knowledge—whether older men were capable of procreation or not; the potentiality of this capacity was sufficient for the usually welcome fitness for marriage. These canonical, civil or private law regulations applied well into the early modern period, in part until the introduction of civil marriage; in Catholic canon law, they still apply in part today [31], (pp. 11–16).

In the early modern period, the legal discourse on age-related male impotence was revived. From 1513 onwards, numerous editions of the humanist jurist André Tiraqueau’s treatise on the secular marriage laws of his home province of Poitou appeared. One of them (no. 6) deals with the desired ages of man and woman at marriage and opposes the union of older men with younger women, but says that it is not the number of years that is decisive but the procreative capacity [32], fol. 104v. Tiraqueau uses this precept for an extensive age-critical excursus on marriage problems of old people and between the generations. Probably most influential for the legal discussion in the 17th and 18th centuries proved to be the monumental canonical treatise by the Spanish Jesuit Tomás Sánchez (written around 1603). Sánchez generally considered impotence to be one of the most frequent impediments to marriage that would be discussed in ecclesiastical tribunals. With regard to age-induced impotence, he first gives a comprehensive overview of the previous canonical positions. According to this, the marriageability of old, even frail people (including women) is generally affirmed, even if they are probably sterile (due to insufficient quality of their semen—he accepted like his predecessors a merely potential *potentia generandi*). In addition, however, Sanchez expresses his own, even further differentiating position: the consummation of marriage (*consummatio*) necessarily includes not only the will to have sexual intercourse and sexual union (penetration), but also insemination, i.e., the passage of semen into the uterus. Therefore, a marriage is only valid if all these circumstances are fulfilled. He thus demands a complete *potentia coeundi* and opposes the opinion of his predecessors. These postulated an “accidental” impotence in old age. In this case, frailty was an *accidens* like a disease, which did not exclude marriage, even if coitus could not be consummated. According to Sánchez, on the other hand, marriage is out of question if it is clear from the outset that *potentia coeundi* is not possible even with medical support. Sánchez thus positions himself with predominantly medical arguments against the admission of a marriage purely for maintenance in old age [33], (vol. 2, pp. 316–317); [34], (pp. 107–110); [35], (pp. 72–74, 105, 109).

## 4. Discussion

Despite the obvious gaps in the information transmission, the five cases presented reveal four essential aspects:

### 4.1. Relevance and Relativisation of Tradition 

Unsurprisingly for the time, both plaintiffs and defenders extensively cite medical and legal authorities from antiquity and early modern times when supporting relevant facts and interpretations from the cases tried (e.g., age limits for cohabitation or procreative capacity, significance of previous or current illnesses). In addition, testimonies of “experienced” persons (*periti*), especially surgeons or midwives, also play a certain role; they are evaluated by the learned medicine as a supplement to the traditional knowledge. This procedure indicates that, at least in Zacchia, medicine still appears as a theoretical discipline (“book medicine”); the Roman physician apparently did not question or examine the people concerned (patients or clients) himself, but merely collected information and evaluated it. In the selection and interpretation of this information, a certain arbitrariness becomes apparent, at least from today’s perspective: in several cases, one could also come to the opposite conclusions based on the facts and events described. In particular, the assessments of the health condition of deceased presumed fathers seem highly speculative even from the contemporary perspective; they arouse the suspicion that the expert unilaterally promoted the interests of one party: An unequivocal vote was obviously desired, and this was achieved more through the use of rhetoric than through a differentiated assessment of facts. 

In none of the five cases was the proof of age impotence based solely on numerical age; rather, expert Zacchia consistently relativised its significance in favour of a corrective that is nowadays referred to as biological age. Yet even the presumed existence of a higher biological age was apparently not sufficient on its own. For in all cases, in addition to the general physical condition, diseases and ailments of the older man are also elaborated as additional, independent parameters for or against the assumption of impotence. This is reminiscent of admission conditions in pre-modern hospitals, which also required not only an older age, but also the specification of further criteria (especially inability to work due to illness or weakness) for the existence of a need for assistance [36], p. 118. However, the connection of biological age or disease with the relativisation of numerical age can hardly be found in the discussions of impotence in the medical literature before Zacchia; rather, physicians there discussed various age limits and detailed pathophysiological causes within the framework of the theory of the humours, which in turn are secondary in the casuistics.

### 4.2. Social Significance of Old-Age Impotence

The surviving cases impressively demonstrate the great importance of sexuality, marriage and procreation for early modern society. As expected, two cases deal with anulment petitions by women in which male impotence played a special role as an argument; for in legal practice it was almost the only means for women to successfully enforce a separation [37], (pp. 79–80); [38], p. 68; [39]. Early modern marriages lasted on average only a relatively short time (10–15 years). Therefore, the rate of remarriage was relatively high, especially among widowers [16], (pp. 23, 70–71). Considerable age differences between spouses were common, which was critically commented on in society [40], (pp. 126–127). The fear was apparently widespread that men could fail in the consummation of a new marriage as a result of *impotentia coeundi* if they entered into a marriage (possibly again) at an older age. A failure to become pregnant as a result of *impotentia generandi*, on the other hand, was not an argument in either court case (in accordance with the opinion of the canonists). 

In two other cases, suspicion of impotence was used as a means of rejecting claims to paternity and alimony (which, however, was only successful once). And finally, impotence could even be used as an argument to reclaim an inter vivos gift. This illustrates in passing the high pecuniary value of family succession and preservation of family property. 

### 4.3. Gender Roles Portrayed in Relation to Sexuality and Procreation 

The processes around age impotence imagine a strict normative role behaviour of women and men in sexual intercourse, which is well known from pre-modern times: According to this, men have the task, and in the context of marriage even the duty (*debitum*), to actively bring about cohabitation and to carry it through to insemination. Married women only have the task or duty to be passively ready for this and not to close themselves off to the men. If they do the latter (as reported in *consilium* 68), there must be special reasons. At least from a legal point of view, lust or satisfaction play no role on the female side. While women can support or even challenge the sexual behaviour of men through their attractiveness, they are in no way obliged to do so; rather, sexually active behaviour attributed to the alleged prostitute Angela is condemned by depriving her children of the recognition of their parentage. These normative gender roles correspond with the Aristotelian conception of procreation, which ascribes to the man the decisive role in procreation, while to the woman the decisive task in the formation of the foetus until birth. Further details of the different doctrines of procreation, especially the one-sex or two-sex theory of anatomy often discussed in gender research [41], are not touched upon in the legal and forensic texts.

This distribution of activity and passivity between the two sexes made it easier at least for virgins to sue their older husbands for impotence in court: if it was clear from their testimony, possibly supported by a vaginal examination, that cohabitation had not taken place, men were in need of proof (cf. *consilium* 68).

### 4.4. Discrepancy between Medical and Legal Criteria of Judgement

In passing, the texts also suggest differences between the two disciplines: The arguments refuted in *consilia* 68 and 75, supported by Aristotle and other authorities, still assume numerically fixed age limits for procreative capacity; they were presumably put forward by lawyers with legal, but not medical, experience. Similar references can be found in the judicial *decisiones* 59 and 100, which, as reasons for judgement, predominantly reflect legal expertise. Zacchia, on the other hand, as already mentioned, represents the concept of biological ageing as a physician. He therefore has got to emphasise that, at least in the case of men, there are actually no fixed age limits—a statement that was rather unsatisfactory from a legal point of view, because fixed ages for certain areas of law (e.g., (in)maturity, conscription) had formed an integral part of at least Roman law since antiquity. Therefore “jurists expressed reservations about the allegedly intrinsic weakness of medicine—it was based on mere conjectures and could never deliver sound and universal knowledge—an accusation that with little change would be made again and again throughout the centuries.” [19], p. 223. According to Zacchia, in order to form a judgement in questions of age impotence, a personal assessment of the age condition by the judges themselves or even a medical expert is therefore additionally necessary. In this way, the parties involved are entering new legal and medical territory, because there is no longer any safeguarding of this assessment by the traditional authorities. In this respect, Zacchia’s expert opinion, despite his many references to authorities, already shows the first signs of medical empiricism, which recognises and takes into account the biological variance between individuals.

## 5. Conclusions

According to the expert Paolo Zacchia, whether old men are impotent and/or infertile is not determined by their numerical age, but by their biological age in connection with individual diseases and pathological conditions. The Roman physician rejects a general determination, even though in his opinion the probability of impotence increases with age. This statement, which is self-evident from today’s perspective, contradicted the age limits for fertility handed down from Greco-Roman antiquity. Against the simple, monocausal explanation with the help of unspecific age-related weakness, Zacchia sets a complex aetiology of male impotence. The latter was already known to scholarly medicine before him, but had not yet been applied to old-age impotence. 

The fact that old-age impotence occurs relatively frequently in the casuistics of the Rota Romana illustrates the social relevance of the topic: marriages and other relationships between the sexes were not infrequently characterised by a large age difference between the partners, among other things because of the high mortality rate and the subsequent new partnerships [16], p. 23; [42]. Traditionally, a finding of impotence was one of the few means of obtaining an annulment of marriage. However, the canonical hurdles for this were particularly high in the case of age impotence, since the Medieval respectively Catholic Church did not recognise *impotentia generandi* as an indication in this case; rather, proof of initial *impotentia coeundi* had to be provided. In addition, age impotence was also relevant in the succession of generations in families, especially in the promotion of generative behaviour, but also in the rejection of undesirable natural offspring.

It follows from all this that old-age impotence and sterility were less a medical than a legal and social issue in the 17th century. This had already been stated by Zacchia’s older contemporary François Ranchin in a short statement [43], p. 483. The fact that Zacchia devoted such attention to this topic from a medical perspective in the main part of his forensic *Quaestiones medicales* is thus not an expression of an early medicalisation of generativity and sexuality. Rather, it reflects the legal need for medical expertise, as it had existed for centuries, at least in the field of family law. However, the canonical discourse became more specific in the 17th century, especially with Sánchez. This required additional medical knowledge with regard to age-related obstacles to marriage: even and especially in the case of older people, knowledge of the exact physical conditions of procreation was more important than ever.

## Data Availability

Not applicable.
